# Biliary Dyskinesia Presenting as Atypical Chest Pain: A Case Report

**DOI:** 10.7759/cureus.92530

**Published:** 2025-09-17

**Authors:** Murali K Manikkavelu, Aryan Kahlon, Mark Salib, John Salib, Frederick Tiesenga

**Affiliations:** 1 General Surgery, Community First Medical Center, Chicago, USA; 2 Medical School, St. George’s University School of Medicine, True Blue, GRD

**Keywords:** atypical chest pain, biliary dyskinesia, cholelithiasis, functional gallbladder disease, gallbladder ejection fraction, gallbladder motility disorder, hida scan, laparoscopic cholecystectomy, non-cardiac chest pain, surgical decision-making

## Abstract

Biliary dyskinesia typically presents with right upper quadrant or epigastric pain, but we describe an atypical case in a 58-year-old man with diabetes who presented with left-sided chest pain radiating to his left arm. His presentation initially suggested a cardiac etiology; however, cardiac evaluation, including stress testing and serial troponins, was negative. CT imaging incidentally revealed gallstones, prompting further investigation. Hepatobiliary scintigraphy subsequently confirmed biliary dyskinesia with a reduced gallbladder ejection fraction of 19%. Although surgical management was recommended, the patient elected to defer cholecystectomy and continue outpatient follow-up. This case highlights the importance of maintaining a broad differential diagnosis when standard evaluations are unrevealing, as biliary pathology may mimic cardiac symptoms.

## Introduction

Biliary dyskinesia is a functional gallbladder disorder characterized by recurrent right upper quadrant or epigastric pain. It presents with a decreased gallbladder ejection fraction on hepatobiliary scintigraphy [1]. The pathophysiology stems from impaired gallbladder contractility, resulting in inadequate bile release and secondary complications such as biliary colic, gallbladder spasm, bile stasis, and eventual cholecystitis [2,3].

The clinical presentation can be heterogeneous and often overlaps with other gastrointestinal and cardiopulmonary conditions. In addition to localized right upper quadrant discomfort, patients may experience nonspecific symptoms such as bloating, nausea, back pain, or even chest pressure, which can mimic cardiac ischemia and misdirect diagnostic efforts [4]. This overlap highlights the importance of maintaining a broad differential when evaluating abdominal or chest pain, especially when initial cardiac or gastrointestinal studies are unrevealing.

Diagnostic evaluation typically begins with transabdominal ultrasound and liver function testing, both of which are frequently normal in biliary dyskinesia [3]. The gold standard for diagnosis remains the hepatobiliary iminodiacetic acid (HIDA) scan with cholecystokinin stimulation, where an ejection fraction of <35% has classically been considered abnormal [5]. Despite its utility, diagnostic thresholds and management guidelines vary considerably across institutions and geographic regions, contributing to the under-recognition of this disorder [6]. Epidemiologic studies suggest biliary dyskinesia may be more prevalent than previously appreciated, with recent estimates reporting a prevalence of up to 21% in females and 8% in males [7].

Although biliary dyskinesia is increasingly recognized, atypical presentations remain underreported and underappreciated. These cases emphasize the importance of considering biliary pathology in patients with unexplained chest or abdominal pain after negative cardiac and gastrointestinal evaluations. The present report describes such an atypical presentation, reinforcing the need for heightened clinical awareness and systematic evaluation of this condition.

## Case presentation

A 58-year-old gentleman with a past medical history of type 2 diabetes mellitus, depression, generalized anxiety disorder, and attention-deficit hyperactivity disorder presented to the emergency department with a history of left-sided chest pain. The pain began the previous day while at rest and persisted through the night. He described the pain as a constant, dull, crampy sensation located near his left axilla and radiating to the left shoulder and arm. Associated symptoms included weakness, paresthesias of the left upper extremity, and intermittent left-sided neck tightness over the past week. The patient rated his pain 2.5 out of 10 in severity, without clear aggravating or relieving factors, and was accompanied by transient anxiety and mild diaphoresis during one episode. In addition, the patient reported intermittent bloating without changes in bowel or urinary habits. He denied shortness of breath, nausea, vomiting, diarrhea, fever, chills, headache, vision changes, abdominal pain, back pain, pelvic pain, recent travel, or sick contacts.

Initial vital signs demonstrated a blood pressure of 185/102 mmHg, heart rate of 77 beats/minute, respiratory rate of 20 breaths/minute, temperature of 97.5°F, oxygen saturation of 96% on room air, and a body mass index of 33.8 kg/m². On physical examination, the patient’s lungs were clear to auscultation bilaterally with unlabored respirations. Cardiac examination revealed a regular rate and rhythm with normal S1 and S2, without murmurs, rubs, or gallops. The abdomen was soft, non-tender, and non-distended with active bowel sounds. No hepatosplenomegaly, masses, or peritoneal signs were appreciated, and there was no documentation of right upper quadrant tenderness or a positive Murphy’s sign.

Laboratory evaluation is summarized in Table 1. Results were notable for uncontrolled diabetes with hyperglycemia (glucose = 321 mg/dL) and an elevated HbA1c of 10.2%. Complete blood count and basic metabolic panel were otherwise unremarkable, and liver function tests were within normal limits. High-sensitivity troponins were negative on two occasions.

**Table 1:** Admission laboratory results. Baseline laboratory studies obtained in the emergency department. Values are presented with corresponding reference ranges. HbA1c = hemoglobin A1c; AST = aspartate aminotransferase; ALT = alanine aminotransferase

Test	Result	Reference range
White blood cells (k/µL)	8.2	4.0–11.0
Hemoglobin (g/dL)	17	13.5–17.5
Platelets (k/µL)	247	150–450
Glucose (mg/dL)	321	70–110
Sodium (mmol/L)	136	135–145
Potassium (mmol/L)	3.9	3.5–5.0
Creatinine (mg/dL)	0.75	0.6–1.3
AST (IU/L)	21	10–40
ALT (IU/L)	38	7–56
Alkaline phosphatase (IU/L)	90	40–129
Total bilirubin (mg/dL)	0.8	0.1–1.2
HbA1c (%)	10.2	<5.7 (normal); ≥6.5 (diabetes)
High-sensitivity troponin (ng/L)	3	<14

**Figure 1:**
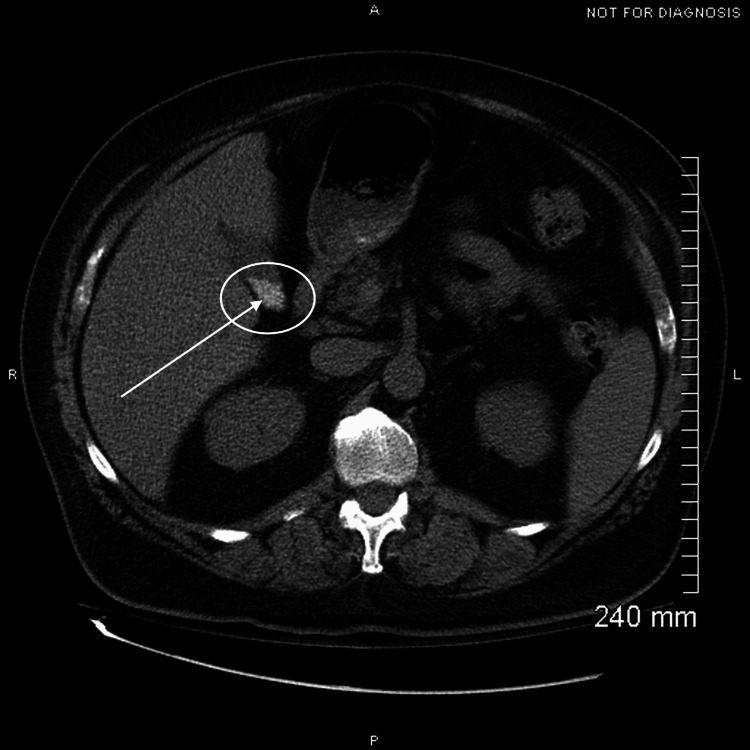
Axial CT of the abdomen/pelvis showing a hyperdense focus within the gallbladder consistent with gallstones. No wall thickening, pericholecystic fluid, or ductal dilation is present.

**Figure 2:**
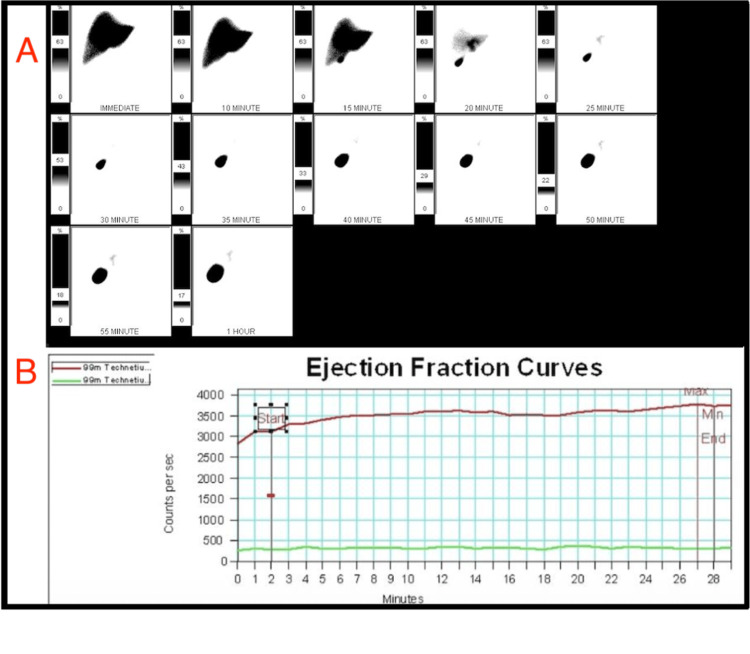
Hepatobiliary scintigraphy with gallbladder ejection fraction curve. (A) Sequential 99mTc-mebrofenin images demonstrate prompt hepatic uptake, excretion into the biliary tree, and gallbladder visualization over 60 minutes. (B) Gallbladder time-activity curve (red) with background subtraction (green). Following CCK stimulation, a minimal decrease in counts was observed, yielding a gallbladder ejection fraction of 19%, consistent with biliary dyskinesia. Tc = technetium; 99mTc = technetium-99m; CCK = cholecystokinin; ROI = region of interest; EF = ejection fraction

A chest X-ray was performed for chest pain and showed no acute cardiopulmonary process. A stress echocardiogram was then performed to assess for myocardial ischemia, which demonstrated normal left ventricular wall motion without evidence of ischemia [8]. A CT angiogram of the chest was performed to evaluate for pulmonary embolism or dissection in the setting of chest pain, but it showed no acute thoracic pathology. After that, a CT angiogram of the abdomen and pelvis was performed to extend the possibility of an abdominal aortic dissection in his workup; however, it incidentally demonstrated cholelithiasis without other acute findings [9] (Figure 1). The following day, further biliary testing was completed, including hepatobiliary scintigraphy with ejection fraction, to assess for functional gallbladder disorder, which revealed gallbladder dyskinesia with an ejection fraction of 19% (Figure 2) [3].

Cardiac and vascular causes were excluded through negative echocardiography and CT angiography findings [8]. Incidental CT angiography of the abdomen and pelvis identified cholelithiasis [9], and hepatobiliary scintigraphy confirmed gallbladder dyskinesia with an ejection fraction of 19%, which is diagnostic when below 35% [3]. The diagnosis was further supported by normal liver function tests, reducing the likelihood of acute cholecystitis or biliary obstruction. This established a diagnosis of functional gallbladder disorder in the presence of gallstones. Laparoscopic cholecystectomy was recommended as definitive management [1]; however, after a discussion of risks and benefits, the patient elected to defer surgery, preferring to seek a second opinion given his perception that he was not experiencing typical gallbladder symptoms.

He was counseled on dietary modification, particularly adherence to a low-fat diet to minimize biliary stimulation, and provided with analgesics for symptom control. He was educated on the importance of recognizing warning signs of complications such as severe right upper quadrant pain, fever, jaundice, or vomiting. Outpatient follow-up with surgery was arranged to allow reassessment of symptoms, repeat imaging if indicated, and reconsideration of surgical intervention should his clinical status change. He was discharged with coordinated outpatient follow-up to ensure ongoing evaluation and management.

## Discussion

This case underscores the diagnostic challenges posed by atypical presentations of biliary dyskinesia, particularly when symptoms mimic more common cardiopulmonary or neurologic conditions [4,8]. Our patient’s left-sided chest pain, radiating to the left arm with associated paresthesia, initially suggested a cardiac or neurologic etiology. Standard laboratory workup, imaging, and functional studies were unremarkable [9], necessitating a broader diagnostic approach. This highlights the need for an adjusted framework in the evaluation of biliary pathologies, especially in underdiagnosed conditions where classic symptoms such as right upper quadrant pain, fever, or abnormal liver function tests may be absent.

The diagnostic evaluation in this case began with exclusion of cardiopulmonary and vascular causes of chest pain through stress echocardiography and CT angiography, both of which were negative for acute ischemia, pulmonary embolism, or dissection. Subsequent CT angiography of the abdomen and pelvis, performed to further assess for abdominal aortic pathology, incidentally revealed cholelithiasis without evidence of acute cholecystitis. To assess for functional biliary pathology, hepatobiliary scintigraphy with cholecystokinin stimulation was obtained, demonstrating a gallbladder ejection fraction of 19%, which is diagnostic for biliary dyskinesia when below 35%. Normal liver function tests further reduced the likelihood of acute cholecystitis, cholangitis, or biliary obstruction. Taken together, these findings supported a diagnosis of functional gallbladder disorder in the setting of gallstones [9].

Functional imaging, specifically hepatobiliary scintigraphy, was pivotal in establishing the diagnosis, revealing a gallbladder ejection fraction of 19%, well below the 35% threshold for biliary dyskinesia [1,3]. While an ejection fraction <35% is generally accepted as diagnostic, outcomes are not limited to this subgroup. In a large series, Alexida and Tiesenga examined patients undergoing laparoscopic cholecystectomy across a broad range of HIDA ejection fractions, including borderline (35-50%) and hyperkinetic (>75%) values. They reported symptomatic improvement rates of 92% for low ejection fraction, 72% for borderline ejection fraction, and 65% for hyperkinetic ejection fraction [5]. This study demonstrates that surgical benefit can extend beyond the traditional threshold, highlighting the variability in defining abnormal gallbladder contractility. These findings suggest that the decision to operate should be guided by clinical presentation and symptom correlation, rather than ejection fraction alone.

Given the central role of the HIDA scan in this case, alternative non-invasive approaches, such as fatty-meal ultrasound, may be considered for patients who wish to avoid radiation exposure [9,10]. This method assesses gallbladder contractility before and after a standardized fatty meal to calculate ejection fraction and may serve as a reasonable preliminary diagnostic tool.

The definitive treatment for gallbladder dyskinesia with or without cholelithiasis is laparoscopic cholecystectomy, particularly in patients presenting with biliary-type pain and an ejection fraction below 35% on hepatobiliary scintigraphy. Surgical management has been shown to provide the greatest likelihood of long-term symptom relief and prevent complications such as recurrent biliary colic, acute cholecystitis, or gallstone pancreatitis. In patients who decline surgery or are poor operative candidates, conservative measures may be employed. These include dietary modification with a low-fat diet, symptomatic pharmacologic therapy such as nonsteroidal anti-inflammatory drugs for biliary pain and antiemetics for nausea, and, in select cases, ursodeoxycholic acid for gallstone dissolution, although its utility is limited and recurrence rates are high. Conservative management is generally reserved for those with atypical or minimal symptoms, significant comorbidities, or patient preference, with careful outpatient follow-up to reassess symptoms and reconsider surgical intervention if clinical status changes [1,10].

In this case, the decision to pursue conservative management was influenced by several patient-specific factors. Although hepatobiliary scintigraphy demonstrated an abnormal gallbladder ejection fraction consistent with biliary dyskinesia, the patient did not endorse classic biliary colic symptoms; instead, the patient presented with atypical left-sided chest and shoulder pain. The absence of right upper quadrant tenderness, nausea, vomiting, or postprandial exacerbation further reduced the likelihood that his symptoms were directly attributable to gallbladder dysfunction. Additionally, normal liver function tests and lack of inflammatory changes on imaging argued against acute cholecystitis or biliary obstruction, decreasing the urgency for surgical intervention. Given this atypical presentation, alongside the patient’s preference to defer surgery and seek a second opinion, conservative outpatient management with dietary modification, symptom monitoring, and coordinated follow-up was considered appropriate. This approach balanced the potential benefits of early surgical intervention against the patient’s autonomy, comorbidities, and uncertainty regarding the symptomatic relevance of his gallbladder findings [11-13].

Overall, this case emphasizes several key teaching points: biliary dyskinesia can present atypically and mimic cardiopulmonary or neurologic conditions; functional imaging is essential when standard labs and imaging are non-diagnostic; and management decisions should balance objective findings with patient preferences. Clinicians should maintain a high index of suspicion for functional gallbladder disorders in atypical presentations and tailor evaluation and management accordingly.

## Conclusions

This case demonstrates the diagnostic challenges posed by atypical presentations of biliary dyskinesia, where classic symptoms, laboratory abnormalities, and initial imaging may be absent. Functional imaging, such as hepatobiliary scintigraphy, was pivotal in establishing the diagnosis in this patient, whose chest pain initially suggested a cardiac or neurologic etiology. Although surgical intervention was indicated, the patient’s decision to defer highlights the importance of shared decision-making and respecting patient autonomy. Notably, patients with borderline or even hyperkinetic ejection fractions have also been shown to benefit from surgery, underscoring that outcomes are not limited to those with severely reduced values. Clinicians should maintain a high index of suspicion for functional gallbladder disorders in atypical presentations and tailor evaluation and management to both objective findings and patient preferences, while considering non-invasive diagnostic alternatives when appropriate.

## References

[1] Abuahmed MY, Wuheb A, Eskandar G (2024). The management of dysfunctional gallbladder disease and the role of laparoscopic cholecystectomy on symptom improvement: a retrospective cohort study. Cureus.

[2] Arshi J, Layfield LJ, Esebua M (2021). Mast cell infiltration and activation in the gallbladder wall: implications for the pathogenesis of functional gallbladder disorder in adult patients. Ann Diagn Pathol.

[3] Flick KF, Soufi M, Sublette CM, Sinsabaugh CA, Colgate CL, Tann M, House MG (2021). Optimal hepatobiliary scintigraphy for gallbladder dyskinesia. Surg Open Sci.

[4] Fass R, Achem SR (2011). Noncardiac chest pain: epidemiology, natural course and pathogenesis. J Neurogastroenterol Motil.

[5] Alexida L, Tiesenga FM (2017). Laparoscopic cholecystectomy for biliary dyskinesia in patient with an extended spectrum of ejection fraction on hepatobiliary iminodiacetic acid scan. Int Arch Integr Med.

[6] Bielefeldt K (2013). Regional differences in hospitalizations and cholecystectomies for biliary dyskinesia. J Neurogastroenterol Motil.

[7] (2025). Radiopaedia. Functional gallbladder disorder. https://radiopaedia.org/articles/functional-gallbladder-disorder.

[8] Kontos MC, Diercks DB, Kirk JD (2010). Emergency department and office-based evaluation of patients with chest pain. Mayo Clin Proc.

[9] Chawla A, Bosco JI, Lim TC, Srinivasan S, Teh HS, Shenoy JN (2015). Imaging of acute cholecystitis and cholecystitis-associated complications in the emergency setting. Singapore Med J.

[10] Spangenberg B, van Rensburg JJ (2018). Fatty meal sonography comparing coconut oil and chocolate bar with full-fat yoghurt as cholecystagogues for gallbladder ejection fractions. SA J Radiol.

[11] McGillicuddy EA, Schuster KM, Barre K (2012). Non-operative management of acute cholecystitis in the elderly. Br J Surg.

[12] Elwyn G, Frosch D, Thomson R (2012). Shared decision making: a model for clinical practice. J Gen Intern Med.

[13] Barry MJ, Edgman-Levitan S (2012). Shared decision making--pinnacle of patient-centered care. N Engl J Med.

